# Non-selective beta blockers inhibit angiosarcoma cell viability and increase progression free- and overall-survival in patients diagnosed with metastatic angiosarcoma

**DOI:** 10.18632/oncoscience.413

**Published:** 2018-04-29

**Authors:** Clarissa N. Amaya, Mariah Perkins, Andres Belmont, Connie Herrera, Arezo Nasrazadani, Alejandro Vargas, Thuraieh Khayou, Alexa Montoya, Yessenia Ballou, Dana Galvan, Alexandria Rivas, Steven Rains, Luv Patel, Vanessa Ortega, Christopher Lopez, William Chow, Erin B. Dickerson, Brad A. Bryan

**Affiliations:** ^1^ Department of Biomedical Sciences, Texas Tech University Health Sciences Center, El Paso, TX, USA; ^2^ Department of Biochemistry, Baylor University, Waco, TX, USA; ^3^ Paul L. Foster School of Medicine, Texas Tech University Health Sciences Center, El Paso, TX, USA; ^4^ Department of Biology, University of Texas, El Paso, TX, USA; ^5^ Mohs Micrographic Surgery and Cutaneous Oncology, San Leandro, CA, USA; ^6^ Department of Veterinary Clinical Sciences, University of Minnesota, St. Paul, MN, USA; ^7^ Masonic Cancer Center, University of Minnesota, Minneapolis, MN, USA

**Keywords:** angiosarcoma, propranolol, beta blocker, sarcoma

## Abstract

Patients with metastatic angiosarcoma undergoing chemotherapy, radiation, and/or surgery experience a median progression free survival of less than 6 months and a median overall survival of less than 12 months. Given the aggressive nature of this cancer, angiosarcoma clinical responses to chemotherapy or targeted therapeutics are generally very poor. Inhibition of beta adrenergic receptor (β-AR) signaling has recently been shown to decrease angiosarcoma tumor cell viability, abrogate tumor growth in mouse models, and decrease proliferation rates in preclinical and clinical settings. In the current study we used cell and animal tumor models to show that β-AR antagonism abrogates mitogenic signaling and reduces angiosarcoma tumor cell viability, and these molecular alterations translated into patient tumors. We demonstrated that non-selective β-AR antagonists are superior to selective β-AR antagonists at inhibiting angiosarcoma cell viability. A prospective analysis of non- selective β-AR antagonists in a single arm clinical study of metastatic angiosarcoma patients revealed that incorporation of either propranolol or carvedilol into patients' treatment regimens leads to a median progression free and overall survival of 9 and 36 months, respectively. These data suggest that incorporation of non-selective β-AR antagonists into existing therapies against metastatic angiosarcoma can enhance clinical outcomes.

## INTRODUCTION

Angiosarcomas are rare vascular tumors of aberrant endothelial histology that exhibit an aggressive and often highly lethal natural course. These tumors can manifest at various sites on the body, but are most frequent in the skin, soft tissue, liver, breast, spleen, bone, or heart [1, 2]. Metastatic angiosarcoma patients face a dismal median progression free survival (PFS) of 3 to 6 months and median overall survival (OS) of 3 to 12 months [3-8]. Overall treatment response rates to chemotherapy or targeted therapeutics in patients with metastatic disease are generally between 10-20% [6, 7, 9-11], and there are no established treatment guidelines for metastatic disease. Because of the poor prognosis of patients with metastatic angiosarcoma, novel therapies that improve outcome are desperately needed.

Several recent studies have reported beta adrenergic receptor (β-AR) expression across diverse tumor types [12-23], and a growing body of literature suggests a role for β-AR signaling in regulating multiple hallmarks of benign and malignant tumors [24-28]. Inhibition of β-AR signaling by receptor antagonists (beta blockers) has become the gold standard treatment for the benign vascular tumor infantile hemangioma [27, 29], in part through disrupting tumor cell proliferation [30]. By translating these findings to malignant angiosarcomas, we showed that propranolol exhibits selective cytotoxicity and tumor suppressive ability against these tumors [21]. Clinical translation of propranolol in combination with chemotherapy drugs has generated overwhelming clinical responses in all reports to date of its use in angiosarcoma patients [31-38]. Among these studies, Pasquier et. al. combined propranolol and the microtubule-targeting agent vinblastine in seven patients with advanced angiosarcomas, and showed that incorporation of propranolol into the chemotherapy regimen leads to a median PFS and median OS at 11 and 16 months, respectively [36]. Collectively, the clinical effectiveness of propranolol against angiosarcomas recently led to its Orphan Drug Designation by the European Medicines Agency (EMA) for use against soft tissue sarcomas.

In addition to vascular tumors, β-AR antagonists have shown preclinical efficacy against a variety of malignant tumors including breast cancer [10, 15, 39-41], neuroblastoma [42], and melanoma [43-48]. Retrospective analyses of patient data have also revealed a strong correlation between reduced tumor progression, metastasis, and mortality, and the use of β-AR antagonists in breast, ovarian, and prostate cancer patients, as well as melanoma patients [15, 49-58]. Prospective clinical studies have reported that propranolol use results in a reduced breast tumor proliferation rates [15], and, when combined with COX-2 inhibitors, β-AR antagonists inhibit multiple cellular and molecular pathways related to metastasis and disease recurrence [59].

In the current study, we evaluated the efficacy of selective (targeting only one β-AR) and non-selective (targeting multiple β-ARs) beta blockers using in vitro angiosarcoma models. We then quantified clinical outcomes of patients with metastatic disease prescribed non-selective beta blockers in combination with standard treatment regimens.

## RESULTS

**Figure 1:**
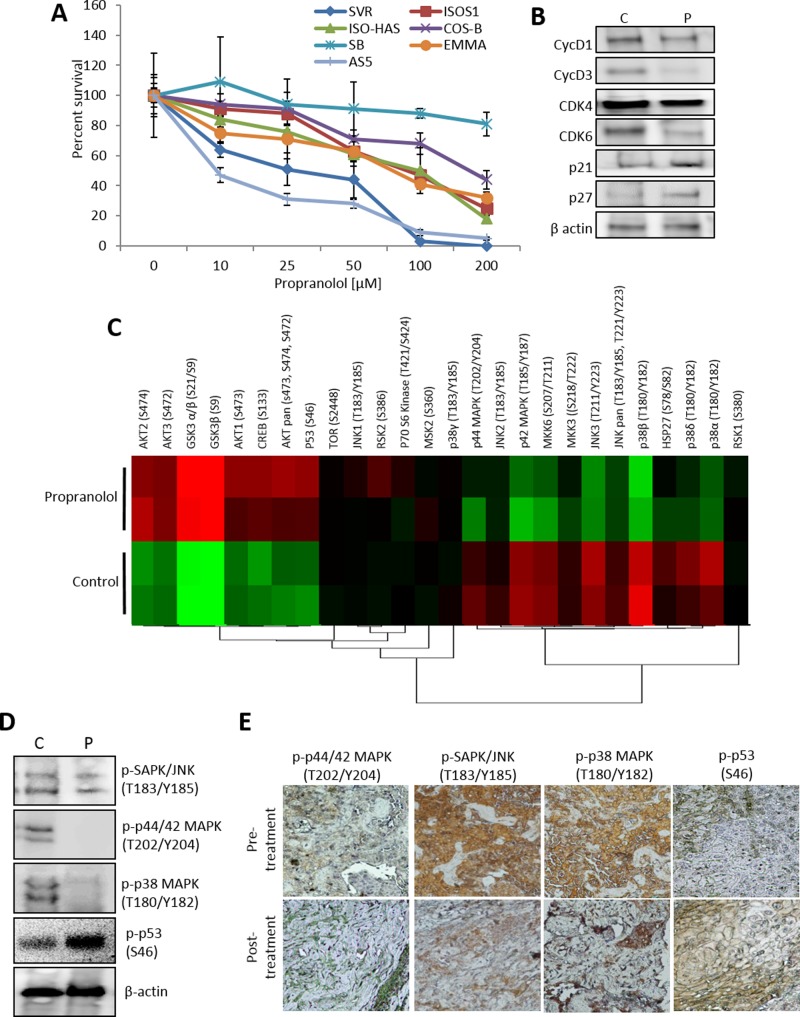
β-AR antagonism inhibits angiosarcoma viability and mitogenic/survival signaling **(A)** Angiosarcoma cell lines were treated with increasing concentrations of the non-selective beta blocker propranolol (0 to 200 µM). Cell viability was measured after 48 hours. **(B)** SVR cells were treated with a DMSO control or 25 µM propranolol for 24 hours. Protein lysates were collected and subjected to immunoblotting to detect levels of key cell cycle regulators. β-Actin was used as a loading control. **(C)** SVR cells were treated with 25 µM propranolol for 1 hour. Protein lysates were subjected to duplicate antibody arrays that simultaneously detected the relative phosphorylation of 24 kinases. The normalized levels of the phosphorylated proteins across the treatments are visualized via a heat map *(red=increased phosphorylation, green=decreased phosphorylation)*. **(D)** Angiosarcoma xenografts were injected with an isotonic saline control or propranolol (15 mg/kg). Cell lysates were collected at 15 minutes post-treatment and subjected to immunoblotting for key mitogenic and survival signaling regulators. β-actin was used as a control to ensure equal loading of the samples. **(E)** Tumor sections from an angiosarcoma patient collected at diagnostic biopsy (before propranolol administration, pre-treatment) and at one week after administration of propranolol (120 mg/day) were analyzed by IHC to determine the levels of p-p44/42 (Thr202/Tyr204), p-SAPK/JNK (Thr183/Tyr185), p-p38 (T180/Y182), and p-p53 (S46). Brown coloration indicates positive antigen staining.

*β-AR antagonism disrupts sarcoma cell viability*. We have previously shown that β-AR antagonists inhibit the viability of angiosarcoma cells and limit tumor progression [21, 32, 36]. We expanded these findings to additional angiosarcoma and hemangiosarcoma cells lines, revealing dose-dependent decreases in cell viability in response to propranolol for all cell lines with the exception of SB-HSA, a hemangiosarcoma line (Figure 1A). Treatment of SVR cells with propranolol reduced the expression of cyclins and cyclin dependent kinases, and increased the expression of cyclin dependent kinase inhibitors (Figure 1B), confirming the impact of propranolol at the molecular level. We then used antibody arrays to assess signaling alterations induced after 1 hour treatment with propranolol, revealing activation of cell stress/survival mediators including AKT and p53, and inhibition of multiple MAPK mediators such as p42, p44, JNK, and p38 (Figure 1C). A subset of these signaling alterations was confirmed in mice bearing SVR angiosarcoma xenografts treated with 15 mg/kg propranolol for 15 minutes. A comparison with tumors from sham treated controls revealed decreased mitogenic p42/44, JNK/SAPK, and p38 phosphorylation and increased apoptotic p53 phosphorylation (Figure 1D). We confirmed these results in tumor samples obtained from an angiosarcoma patient taken before and one week after treatment with propranolol monotherapy. Evaluation of the expression of levels of phosphorylated p44/42, JNK/SAPK, and p38, as well as the apoptotic regulator p53 by IHC showed the levels of all three mitogenic regulators were markedly reduced following propranolol, while the level of phosphorylated p53 was increased (Figure 1E).

**Figure 2:**
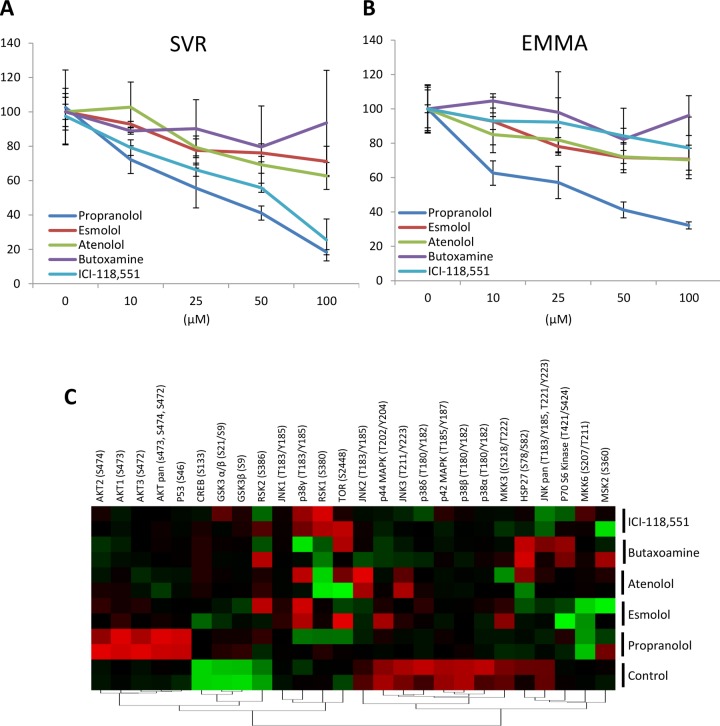
Non-selective β-AR antagonists reduce angiosarcoma cell viability more effectively than selective antagonists (A & B) Two angiosarcoma cell lines, SVR and Emma, were treated with equimolar (25 µM) combinations of propranolol (β1-AR and β2-AR antagonist), esmolol (β1-AR selective antagonist), atenolol (β1-AR selective antagonist), butoxamine (β2-AR selective antagonist), or ICI-118,551 (β2-AR selective antagonist). Cell viability was assessed after 48 hours of treatment. (C) SVR cells were treated with equimolar (25 µM) concentrations of propranolol, esmolol, atenolol, butoxamine, or ICI-118,551 for 1 hour. Protein lysates were analyzed in duplicate using an antibody array that simultaneously detects the relative phosphorylation of 24 kinases. The normalized levels of the phosphorylated proteins across the treatments were visualized via a heat map *(red=increased phosphorylation, green=decreased phosphorylation)*.

*Non-selective β-AR antagonists are superior to selective β-AR antagonists for inhibiting angiosarcoma viability*. Propranolol is a non-selective β-AR antagonist that classically inhibits both β1-AR and β2-AR. Our lab and others have previously shown that non-selective β-AR antagonists are superior to selective β-AR antagonists with regard to decreasing tumor cell proliferation and enhancing clinical outcome in breast, liver, and ovarian cancer [15, 57, 58]. To determine if a similar outcome could be observed in angiosarcoma models, we evaluated the viability of two angiosarcoma cell lines following treatment with propranolol, β1-AR selective antagonists (esmolol and atenolol), or β2-AR selective antagonists (butoxamine and ICI-118,551). β3-AR selective antagonists were not evaluated since selective antagonists targeting this receptor are not clinically available to patients. While moderate decreases in cell viability were observed following treatment of the angiosarcoma cells with the selective receptor antagonists, none of the selective antagonists were as effective as propranolol (Figure 2A & 2B). To determine if the observed difference was due to activation or inhibition of different cell signaling pathways, SVR cells were treated with non- selective or selective antagonists for 1 hour and mitogenic and survival signaling changes were evaluated using antibody arrays. Compared to control samples, the majority of the protein phosphorylation events were consistent between propranolol and the selective β-AR antagonists. The major exceptions to this involved propranolol-mediated upregulation of AKT and p53 phosphorylation, which did not occur upon treatment with the selective β–AR antagonists (Figure 2C).

**Figure 3:**
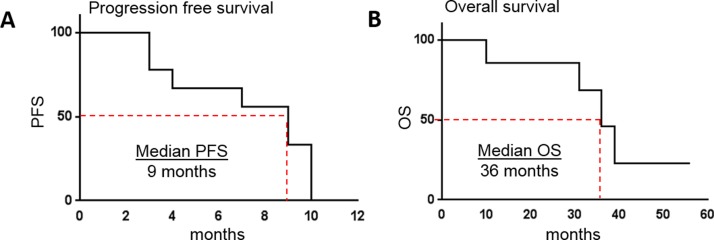
Non-selective β-AR antagonists increase PSF and OS in patients with metastatic angiosarcoma Patients with an initial diagnosis of metastatic angiosarcoma were prescribed the non-selective beta blockers propranolol (n=8) or carvedilol (n=1) in addition to their standard anti-cancer treatment regimen. PFS **(A)** and OS **(B)** were tracked between the years of 2013 to 2017.

*Non-selective β-AR antagonists extend progression free and overall survival in patients with metastatic angiosarcoma*. Patients with an initial diagnosis of metastatic angiosarcoma were prescribed the non-selective beta blockers propranolol or carvedilol in addition to their anti-cancer treatment regimen. Six women and three men were included in this study, with a median age of 53.7 (range=34-75). Each patient was given propranolol (20 to 100 mg per day; n=8) or carvedilol (6.25 mg per day; n=1) in combination with treatment protocols, which varied by treating oncologist (Table 1). Patients treated with non-selective β-AR antagonists were chosen for this study based on the stronger efficacy observed in our preclinical models of angiosarcoma (Figures 1 & 2). A single patient declined chemotherapy or radiation, and was only prescribed propranolol and supportive care. All patients were monitored between the years of 2013 to 2017. PFS and OS rates were improved in patients taking β-AR antagonists relative to the historical controls [4]. Specifically, administration of β−AR antagonists in combination with anti-cancer therapies resulted in a median PFS of 9 months verses previous reports of 3 to 6 months (Figure 3A), and a median OS of 36 months verses documented reports of 12 months (Figure 3B). The correlation coefficient between daily dose of propranolol and PFS was 0.25, suggesting a very weak/absent relationship between the doses of propranolol used in this study and clinical outcomes.

**Table 1:** Clinical information for metastatic angiosarcoma patients

Sex	Primary	Metastases	Treatment	Antagonist	Dose/day
F	Breast	Skull	Doxorubicin, Cyclophosphamide, Radiotherapy	Propranolol	60 mg
M	Spleen	Bone marrow, liver	Doxorubicin, Radiotherapy, Surgery	Carvedilol	6.25 mg
F	Breast	Lymph nodes, chest cavity	Paclitaxel, Ifosfamide, Carboplatin	Propranolol	40 mg
M	Pleura	Lymph nodes, Bone	Doxorubicin	Propranolol	100 mg
F	Breast	Lungs	Paclitaxel, Cyclophosphamide	Propranolol	60 mg
M	Liver	Spleen, Lungs	Doxorubicin, Radiotherapy	Propranolol	40 mg
F	Breast	Liver	Doxorubicin, Gemcitabine	Propranolol	20 mg
F	Breast	Brain, Bone, Lungs, Maxillary Sinus, Thigh	Doxorubicin, Ifosfamide, Paclitaxel, Gemcitabine, Radiotherapy	Propranolol	40 mg
F	Cardiac	Lungs, Liver	None	Propranolol	40 mg

## DISCUSSION

The data presented in this study demonstrate that non-selective β-AR antagonists effectively inhibit tumor cell viability and mitogenic signaling in angiosarcomas. Furthermore, incorporation of non-selective β-AR antagonists into the standard treatment of patients with metastatic angiosarcoma substantially increased PFS and OS compared to historical controls.

The influence of β-AR signaling on non-diseased and tumor cell proliferation rates was reported in the literature more than half a century ago [28]. We have previously reported in angiosarcoma and breast cancer patient case studies that neoadjuvant administration of propranolol alone reduced the tumor proliferative index based on quantification of tumor Ki-67 levels [15, 32]. With the exception of the propranolol-resistant SB cell line, our current data demonstrates that concentrations of propranolol as low as 10 µM reduces angiosarcoma cell viability between 6% to 53% compared to controls, with increasingly stronger anti-proliferative affects observed at elevated concentrations of the drug. We validated these findings at the molecular level using in vitro assays, a xenograft tumor model, and patient tumor analysis to demonstrate that propranolol consistently reduces mitogenic signaling and increases apoptotic signaling in angiosarcomas. While our results show that β-AR antagonists disrupt tumor cell function, the dramatic anti-tumor responses observed following administration of β-AR antagonists in angiosarcoma patients may also be due, at least in part, to effects on cells within the tumor stroma. For example, β-AR stimulation increases cellular transformation of fibroblast cells in soft agar [74], suggesting that catecholamine signaling could directly influence tumor stromal behavior. β-AR antagonists also potentiate the anti-angiogenic effects of chemotherapy agents [36] and suppress cell cycle progression and chemotactic motility in endothelial cells [75], suggesting an anti-angiogenic function for these drugs. Catecholamine stimulation of β-ARs disrupts immune cell differentiation, impairs the cytotoxicity of natural killer cells and expansion of memory CD8+ T-cells, and alters T-helper cell responses [76-83]. Recently, propranolol has been shown to influence the activity of the immune system where it facilities the conversion of tumors to an immunologically active tumor microenvironment with increased T-cell infiltration and decreased immunosuppressive PD1 expression [44, 84], suggesting that adrenergic antagonists may be an effective adjuvant to immunotherapy.

Retrospective studies in breast, ovarian, and liver cancer suggest that non-selective β−AR antagonists such as propranolol are more effective against tumors than selective receptor antagonists, which target single β-AR receptors [15, 57, 58]. The current data in angiosarcoma validates previous findings from other cancer types by showing that propranolol more effectively reduces tumor cell viability than β1- or β2-AR-selective antagonists. At the molecular level, non-selective and selective antagonists similarly affect post-translational modifications of mitogenic regulators; however, only propranolol induced phosphorylation of AKT and p53 proteins, suggesting a differential regulation of survival/apoptotic proteins between receptor-selective and non- selective drugs. While all three β-AR receptors are expressed across a variety of cancer types [23], including angiosarcoma [30, 32], the individual contribution each receptor makes to the overall oncogenic processes is not known. It is possible that selective antagonism of a single β-AR is compensated by other expressed β-ARs, thus explaining the insufficiency of selective antagonists to recapitulate the anti-proliferative effects of non-selective antagonists.

The data presented in this study revealed that incorporation of non-selective β-AR antagonism into standard treatment regimens increased PFS and OS from approximately 3 to 6 months and 24 to 33 months, respectively, relative to reported results from several key clinical studies that incorporated only chemotherapy, radiation, and/or surgery [3-8]. The efficacy reported in the current study corroborates data previously published by Pasquier and colleagues showing increased PFS and OS for angiosarcoma patients taking propranolol in combination with chemotherapy [36]. We did not observe a significant correlation between the dose of antagonist and clinical outcome based on PFS, therefore future studies with larger patient cohorts will be needed to optimize dosage. While propranolol led to efficacy when combined with other treatment modalities, we were very surprised to see that when used as a single agent propranolol resulted in sustained disease regression for a patient who declined chemotherapy, radiation, and surgery. While this single instance in no way suggests that patients should opt for adrenergic antagonists in lieu of other established treatment options against metastatic angiosarcoma, this limited observation raises the question if these drugs could be used to extend survival in patients who decline standard treatment and opt for only supportive care.

## MATERIALS AND METHODS

*Cell culture and chemicals.* SVR angiosarcoma [60, 61] (ATCC #CRL-2280) and EMMA hemangiosarcoma cells [62] were grown in Dulbecco's Modified Eagle Medium (DMEM) supplemented with 10% fetal bovine serum (FBS) and penicillin/streptomycin (P/S). COSB hemangiosarcoma [63], SB-HSA hemangiosarcoma cells [64], ISOS-1 angiosarcoma [65], ISO-HAS angiosarcoma [66], and AS5 angiosarcoma cells [67] were grown in EBM-2 Basal Medium (Lonza #CC-3156) supplemented with EGM2 Bulletkit (Lonza #CC-4176). All cells were cultured in 37oC water-jacketed CO2 incubators maintained for gas, temperature, and humidity. The non-selective β-AR antagonist propranolol, the β1- AR selective antagonist esmolol, the β1-AR selective antagonist atenolol, the β2-AR selective antagonist butoxamine, and the β2-AR selective antagonist ICI- 118,551 were used at the concentration and timing as indicated for each experiment.

*Viability assays*. To measure viability, cells were plated in 24 well plates at 0.3 x 106 cells/well, treated as indicated for each experiment, and cell viability was monitored over a 48 hour time course using time lapse microscopy with a Nikon Biostation CT robot. Cell number was manually counted at t=0 hours and t=48 hours. The difference in cell number was used to reflect the viability change for each condition. At least three biological replicates were performed for every cell line, with at least four technical replicates per assay. Data is presented as the average of the technical replicates for a single biological replicate.

*Immunoblotting*. Lysates from cell culture or tumor tissue were collected as indicated for each experiment, subjected to SDS-PAGE, and transferred to polyvinylidene fluoride membranes using the Trans-Blot Turbo Transfer System (Bio-Rad). Membranes were blocked in tris buffered saline plus 3% bovine serum albumin and 0.05% Tween-20, and incubated with the following antibodies as indicated for each experiment: anti-phospho-p38 (T180/Y182) (Cell Signaling #4511), anti-phospho-SAPK/JNK (T183/Y185) (Cell Signaling #4668), anti-phospho-p44/42 (T202/Y204) (Cell Signaling #4370), anti-phospho-p53 (S46) (Cell Signaling #2521), anti-cyclin D1 (Cell Signaling #2978), anti-cyclin D3 (Cell Signaling #2936), anti-CDK4 (Cell Signaling #12790), anti-CDK6 (Cell Signaling #13331), anti-p21 (Cell Signaling #2947), anti-p27 (Cell Signaling #3686), and anti-β actin (Santa Cruz Biotech #sc8432). Each primary antibody was detected with an appropriate 1:1000 HRP-conjugated secondary antibody, subjected to Supersignal West Dura Extended Duration Substrate (ThermoFisher Scientific #34075), and digitally captured using a GE Image Quant Las4000 imaging system.

*Antibody array*. The Phospho-Mitogen-Activated Protein Kinase (MAPK) Antibody Array (R&D Systems #ARY002B) was performed according to the manufacturer's instructions using SVR cell lysates. Densitometry of each antibody array signal was performed using ImageJ software. Reference spots on each array were used to normalize the pixel densities. The numerical protein expression data was normalized, and centroid linkage based on an uncentered correlation similarity metric was performed using Gene Cluster 3.0 software. Heatmaps were generated using JavaTreeView software.

*Animal models.* Animal experiments were performed in accordance to Texas Tech University Health Sciences Center Institutional Animal Care and Use Committee regulations for the care and use of animals in experimental procedures, and all efforts were made to minimize suffering. Mice were housed 4 per cage in a temperature-controlled animal facility on a 12h-12h light-dark cycle. Animals had free access to mouse chow and water. Xenograft angiosarcoma tumor models were generated via subcutaneous injection of 1x105 SVR cells into the dorsolateral flank of 6 week old male J/Nu mice (N=5 mice per experimental condition). Tumors were allowed to grow until ~1cm3, and injected with saline sham or 15 mg/kg propranolol for 15 minutes. Tumor tissue was collected and immediately flash frozen in liquid nitrogen. Tissue lysates were pooled prior to use in immunoblotting.

*Immunohistochemistry (IHC)*. IHC was performed on tumor tissue samples collected from an angiosarcoma patient before and after one week of treatment with propranolol [32]. The following antibodies were used: anti-p-p44/42 (Thr202/Tyr204) (Cell Signaling #4370), anti-p-SAPK/JNK (Thr183/Tyr185) (Cell Signaling #4668), anti-p-p38 (T180/Y182) (Abcam #4822), and anti-phospho-p53 (S46) (Cell Signaling #2521). Antigenicity was detected using the mouse and rabbit Specific HRP/DAB (ABC) Detection IHC Kit (Abcam #ab64264) according to the manufacturer's protocol.

*Clinical study*. This clinical study was approved by the Texas Tech University Health Sciences Center Institutional Review Board (IRB# E17109). As angiosarcoma is a very rare tumor and most individual clinical sites do not see enough patients with this tumor type to accommodate a clinical trial, we recruited nine angiosarcoma patients (six women and three men) with a diagnosis of metastatic disease through the Angiosarcoma Awareness patient support group on Facebook. The median age for this cohort was 55 years with a range from 34 to 75 years. Similar recruitment strategies using social media have been effective for a number of rare diseases where any single treatment center would lack the patient volume necessary to conduct a clinical study [68-73]. PFS and OS were obtained for each patient, and the data were compared to historical controls (25 years of data describing clinical outcomes in angiosarcoma patients treated at the Memorial Sloan Kettering Cancer Center) [4]. Kaplan-Meier plots were generated and statistical analysis was performed using GraphPad Prism 7.03. The Pearson's correlation coefficient for PFS and dose of propranolol was calculated using Microsoft Excel.

## CONCLUSION

Due to the poor prognosis of metastatic angiosarcoma patients, many are faced with the decision to undergo chemotherapy, radiation, and/or surgery or simply accept supportive care—choices that generally lead to similar outcomes. The data presented in this study suggest that incorporation of non-selective β-AR antagonists into a variety of standard treatment regimens enhances PFS and OS in patients with metastatic angiosarcoma. Use of inexpensive and relatively safe non-selective β-AR antagonists along with established treatments against angiosarcoma could improve prognoses for metastatic patients.
